# Improved Dispersion of Bacterial Cellulose Fibers for the Reinforcement of Paper Made from Recycled Fibers

**DOI:** 10.3390/nano9010058

**Published:** 2019-01-04

**Authors:** Zhouyang Xiang, Jie Zhang, Qingguo Liu, Yong Chen, Jun Li, Fachuang Lu

**Affiliations:** 1 State Key Laboratory of Pulp and Paper Engineering, South China University of Technology, Guangzhou 510640, China; fezyxiang@scut.edu.cn (Z.X.); zhangjie@brunp.com.cn (J.Z.); ppjunli@scut.edu.cn (J.L.); 2 Nanjing High Tech University Biological Technology Research Institute Co., Ltd., Nanjing 211899, China; liuqingguo@njiwb.com (Q.L.); chenyong@njiwb.com (Y.C.); 3 Guangdong Engineering Research Center for Green Fine Chemicals, Guangzhou 510640, China

**Keywords:** bacterial cellulose, dispersion, recycled fiber, reinforcement, tensile strength

## Abstract

Bacterial cellulose (BC) can be used to improve the physical properties of paper. However, previous studies have showed that the effectiveness of this improvement is impaired by the agglomeration of the disintegrated BC fibers. Effective dispersion of BC fibers is important to their reinforcing effects to paper products, especially those made of recycled fibers. In this study, carboxymethyl cellulose, xylan, glucomannan, cationized starch, and polyethylene oxide were used to improve the dispersion of BC fibers. With dispersed BC fibers, the paper made of recycled fiber showed improved dry tensile strength. The best improvement in dry tensile index was 4.2 N·m/g or 12.7% up, which was obtained by adding BC fibers dispersed with glucomannan. Glucomannan had the highest adsorption onto BC fibers, i.e., 750 mg/g at 1000 mg/L concentration, leading to the best colloidal stability of BC fiber suspension that had no aggregation in 50 min at 0.1 weight ratio of glucomannan to BC. TEMPO-mediated oxidation of BC was effective in improving its colloidal stability, but not effective in improving the ability of BC fiber to enhance paper dry tensile index while the wet tensile index was improved from 0.89 N·m/g to 1.59 N·m/g, i.e., ~80% improvement.

## 1. Introduction

Due to the low-carbon environmental protection concept, a large proportion of paper and paperboard produced are recycled every year. In 2014, approximately 60% of the raw materials for pulp and paper industries were fibers recycled from waste paper and paperboard globally [1]. However, paper or paperboard made of recycled fibers generally had inferior physical properties compared to those made of virgin fibers [2,3,4]. Consequently, effective reinforcing agents are used for processing recycled fibers into quality paper products. In addition, paper with excessive inorganic fillers for special surface properties, e.g., fire retardant, also requires reinforcement [5]. Nano-cellulose, due to their structural similarity, compatibility and affinity to pulp fibers, as well as their high mechanical strength, was used to reinforce paper made from recycled fibers [3,6,7,8] or paper with excessive inorganic fillers [5]. 

Bacterial cellulose (BC) is a special type of nano-cellulose secreted in vitro by bacteria, of which the most studied species is *Gluconacetobacter xylinus* [9]. BC has the same chemical structure as plant-based cellulose and has a higher crystallinity and degree of polymerization [10,11]. The microstructure of BC is also different from plant-based cellulose. BC does not have a macrofibril structure. The BC microfibrils are 10–100 nm in diameter and interlace with each other forming a fine net structure [12,13,14,15], leading to a high specific surface area. When BC pellicles are disintegrated into small fragments or fibers, abundant free hydroxyls will be released. Being used as reinforcing agents during paper-making, the BC fibers can bridge between fibers and improve the physical properties of papers. Our previous studies have already shown that the addition of BC lower than 1% of paper dry weight can effectively improve the tensile strength of paper produced from some high-quality fibers, such as softwood pulp, hardwood pulp, sugarcane bagasse pulp, and bamboo pulp; these fibers are more fibrillated and, thus, have more surface hydrogen bonding sites for BC fibers bridging between [12]. However, the reinforcing effect of BC on recycled fiber paper was limited [12]. Recycled fibers suffer from fiber hornification causing less surface hydrogen bonding sites [3,6,7,12]. Nevertheless, BC fibers with abundant free hydroxyls should have been able to increase the total numbers of hydrogen bonding sites within the recycled fiber matrix and thus the paper strength [3,6,7]. However, BC fibers aggregate with themselves instead of being evenly distributed within the fiber matrix, causing little improvements in the tensile strength of paper produced from recycled fibers [12]. The agglomeration was also a problem when using nano-cellulose, e.g., cellulose nanofibers (CNF) and nanocrystals (CNC), as reinforcing agents for recycled fiber paper [3]. 

In addition to the mixing of nano-cellulose with plant fibers during sheet forming, the coating of inorganic nanoparticles onto paper surface may also be an effective method to reinforce paper. The coating of Mg(OH)_2_ nanoparticles [16] and Halloysite (Al_2_Si_2_O_5_(OH)_4_·2H_2_O) nanotubes [17,18] onto paper surface can improve paper tensile strength by 40–50%. The dispersion and stabilization of those inorganic nanoparticles were vital to their reinforcing effects; e.g., the Mg(OH)_2_ nanoparticles were stabilized trimethylsilyl cellulose [16] and the halloysite nanotubes were stabilized by hydroxypropylcellulose [17,18]. 

Consequently, the dispersion or stabilization of nano-materials was one of the key factors in determining their effects in paper reinforcement. This study is to explore the effective dispersion or stabilization of BC fibers so that their reinforcing effects on paper made from recycled fibers could be improved. Two types of stabilization mechanisms, i.e., steric and electrostatic repulsions, are often used to explain the dispersion or the colloidal stability of cellulose nanofibers or nanocrystals suspended in water [2]. A good dispersion or colloidal stability can be reached by non-covalently adsorbing or covalently grafting macromolecules to the fiber surface, which prevents the fibers from aggregating due to steric hindrance [19]. Many natural polymers or macromolecules are water soluble and have good adhesion to cellulose fibers such as xyloglucan, β-d-glucan, carboxymethyl cellulose (CMC), etc. [19,20]. Having good adhesion to cellulose fibers, xyloglucan had been proved to disperse cellulose nanocrystals well [19,21]. Adding CMC to cellulose fiber suspension led to better dispersion than adding xyloglucan, which may be due to the surface charges of CMC [19]. Bulky macromolecules or polymer chains, for instance, poly(ethylene oxide) or poly(phenylene oxide), can also be grafted onto cellulose fiber (nanocrystalline cellulose) surfaces leading to the formation of well-dispersed and stable colloidal aqueous suspension [22,23]. The hydroxyls on cellulose nanofiber surface may be chemically modified forming charged groups on surface so that the electrostatic repulsion effects prevent such fibers from aggregating [24]. Cellulose nanofibers or nanocrystals produced by TEMPO-mediated oxidation or concentrated H_2_SO_4_ treatment would have their surfaces modified with negative charges, which may naturally be good for their dispersion [2]. However, for general cellulose nanofibers, surface modification with anionic agents is usually required to obtain better dispersions. Meanwhile, cationic surface modification can also improve the dispersion of cellulose fibers [25]. Cellulose nanofibers can be grafted with glycidyltrimethylammonium chloride (GTMAC) [25] or epoxypropyltrimethylammonium chloride [26] to improve their dispersions. 

In addition to effective dispersion, the increased retention of nano-cellulose during paper forming may further improve the paper strength. With addition of cationic macromolecules, e.g., chitosan and cationic polyacrylamide, CNC and CNF can more effectively improve the strength of paper produced from recycled fibers [3]. The cationic modification of BC fibers increased their retention in paper and, thus, further improved the strength of paper made of sugarcane bagasse pulp [13]. However, BC fiber retention is not a problem for recycled fiber. Our previous study has shown that the BC fiber retention rate can reach 95% in recycled fibers and was much higher than in other high quality fibers, due to smaller fiber size and larger numbers of fiber fines for recycled fibers [12]. It states again that BC fiber aggregation is a key factor preventing its effective reinforcement to paper made of recycled fibers.

In this study, natural or modified polysaccharides such as xylan, carboxymethyl cellulose, glucomannan, and polyethylene oxide were used as additives in order to improve the dispersion of BC fibers through steric repulsions. BC fibers were also oxidized through TEMPO-mediated oxidation in order to generate negative surface charges so that BC fibers dispersion would be improved through electrostatic repulsion. At the last, the dispersed BC fibers were used to reinforce paper produced from recycled fibers and mechanisms involved in various methods for dispersing BC fibers were also investigated.

## 2. Materials and Methods

### 2.1. Materials 

Recycled fiber pulp (RFP), made from old newspaper (ONG) and old magazine (OMG), was obtained from Guangzhou Paper Co., Ltd. (Guangzhou, China) with a Canadian Standard Freeness (CSF) of 270 mL; the pulps were kept at moisture content of 90% at 4 °C and were used within 30 days of preparation. CMC-I (Mw = 90,000, DS = 0.7), CMC-II (Mw = 250,000, DS = 1.2), CMC-III (Mw = 250,000, DS = 0.7), nonionic polyethylene oxide (PEO, average Mw ~4,000,000) and sodium hypochlorite solution (6–14% active chlorine basis) were purchased from Shanghai Macklin Biochemical Co., Ltd. (Shanghai, China). Xylan (from sugarcane bagasse, Mw = 30,000) was purchased from Shanghai Yuanye Biochemical Co., Ltd. (Shanghai, China). Glucomannan (from konjac) was purchased from Hubei Yizhi Konjac Biotechnology Co., Ltd. (Hubei, China). Cationized starch (DS = 0.025), which was produced from (3-chloro-2-hydroxypropyl)trimethylammonium chloride modified corn starch, were provided by Guangzhou Paper Co., Ltd. (Guangzhou, China). All other chemicals used are of analytical grade.

### 2.2. Preparation of Bacterial Cellulose (BC)

*Gluconacetobacter xylinus* ATCC23767 was obtained from Nanjing High Tech University Biological Technology Research Institute Co., Ltd. (Nanjing, China) and used to produce the bacterial cellulose (BC) pellicles. Static fermentation method was used to produce the BC pellicles. Detailed preparation methods and characterizations of the BC pellicles were shown in Xiang et al. [12,13].

### 2.3. BC Fiber Dispersion Evaluation 

Stock solutions (2 g/L) of CMC-I, xylan, cationic starch, glucomannan, and nonionic PEO were prepared. Approximately 5 g (corresponding to 0.075 g dry weight) of wet BC pellicles (MC = 98.5%) or oxidized-BC were cut into small pieces and placed into a lab blender (SKG-1246, Foshan, China). Given amounts (corresponding to give the weight ratio of macromolecules/BC of 0, 0.05, 0.1, 0.25, and 0.5) of the macromolecule stock solutions were added. Deionized (DI) water was then added to make the mixture volume to 250 mL. The mixture was blended by the lab blender for 2 min to prepare the BC fiber suspension with a BC fiber concentration of 0.3 g/L (dry matter). The BC fiber suspension was poured immediately into a 250 mL-cylinder. The freshly prepared suspensions with BC fiber well dispersed was white and non-transparent in the 250 mL-cylinder. With time, the aggregation of BC fibers resulted in the shortening of the non-transparent portion, which were recorded with time.

For zeta potential measurement, particle concentration in the suspension should be kept between 10^−5^ and 10^−2^ volume fraction [27]. The freshly prepared BC fiber suspensions were diluted 10 times with DI water to a BC fiber concentration of 0.03 g/L (dry matter). The pH value was ~7.0 for all the solutions. Approximately 1.5 mL of the diluted fiber suspensions were then poured into the sample cell and the zeta potential was measured through a nanoparticle analyzer (Horiba SZ-100Z, Kyoto, Japan). The dynamic (absolute) viscosities of the solutions of different dispersants were determined at 25 °C by using a rotational viscometer (Brookfield DV-II HE, Middleboro, MA, USA) at a rotational speed of 250 rpm; the solution concentration was set to 0.15 g/L (dry matter), which was the same as the concentration of the dispersants in BC fiber suspensions when weight ratio of macromolecules/BC was 0.5.

### 2.4. Adsorption of Macromolecules onto BC Fibers 

Stock solutions of CMC-I, xylan, cationic starch, glucomannan, and nonionic PEO with various concentrations (200 mg/L, 600 mg/L, and 1000 mg/L) were prepared. Approximately 1 g of wet BC pellicle (MC = 98.5%) was disintegrated in 250 mL DI water by the lab blender in instant mode three times. The disintegrated BC and 30 mL of the stock solution was added into a conical flask. The mixture has a pH of ~7.0 and was stirred at 350 rpm for 2 h. The BC was then filtered and washed through a sand-cored funnel (pore size 30–50 μm). The filtrate was oven dried and the increment of weight was the macromolecules that did not adsorbed on BC. 

### 2.5. The TEMPO-Mediated Oxidation of BC Fibers 

The TEMPO-mediated oxidation of BC fibers was conducted according to Saito et al. [28] with minor modifications. Approximately 20 g of wet BC pellicle (MC = 98.5%) was disintegrated by the lab blender in water in instant mode for three times and the water was filtered out through a sand-cored funnel (pore size 30–50 μm). The disintegrated BC fibers were suspended in water (30 mL) containing TEMPO (0.008 g, 0.05 mmol) and sodium bromide (0.05 g, 0.5 mmol). Into the BC fiber suspension, 0.5 mL, 1 mL, and 2 mL of the NaClO solution (6–14% active chlorine basis) were added. The pH was adjusted 10 by the addition of 0.1 M HCl. The TEMPO-mediated oxidation was continued at room temperature by stirring at 350 rpm for 3 h. During the reaction, the pH was maintained at 10 by adding 0.5 M NaOH. The oxidized BC fibers were filtered through a sand-cored funnel (pore size 30–50 μm) and washed with DI water until pH neutral. The samples were denoted OBC-0.5, OBC-1, and OBC-2, respectively, according to the volume of NaClO solution added to the oxidation process, e.g., 0.5 mL, 1 mL, and 2 mL.

### 2.6. Paper Handsheet Preparation and Characterization 

BC membranes were mixed with stock solutions of CMC, xylan, cationic starch, glucomannan, or nonionic PEO and mechanically disintegrated by a lab blender (SKG-1246, Foshan, China) for 5 min. The recycled fiber pulps and the disintegrated BC fibers were mixed with 1% ratio (proportion of BC based on the total fiber dry weight) and dispersed with a standard pulp-disintegrator at a consistency of 1% (*m*/*m*) for 15,000 r. The hand sheets were made from the mixed pulp through a standard sheet former (Labtech 200-1, Laval, QC, Canada). The grammage of each sheet was maintained at 70 g/m^2^ (dry weight) level. The sheets were dried and equilibrated for 24 h at 23 °C and 50% humidity prior to physical tests. The dry tensile index and air permeability of the sheets were tested according to TAPPI standards (T494 and T251 wd-96, respectively). For wet tensile strength measurement, the middle part of the specimens was soaked in water for 8 s and the specimens were tested immediately for the tensile index. Paper sheet surface morphologies were evaluated using SEM (Zeiss Evo-18, Munich, Germany).

## 3. Results and Discussion

### 3.1. BC Fiber Dispersion and Reinforcement of Paper Handsheets

**Figure 1:**
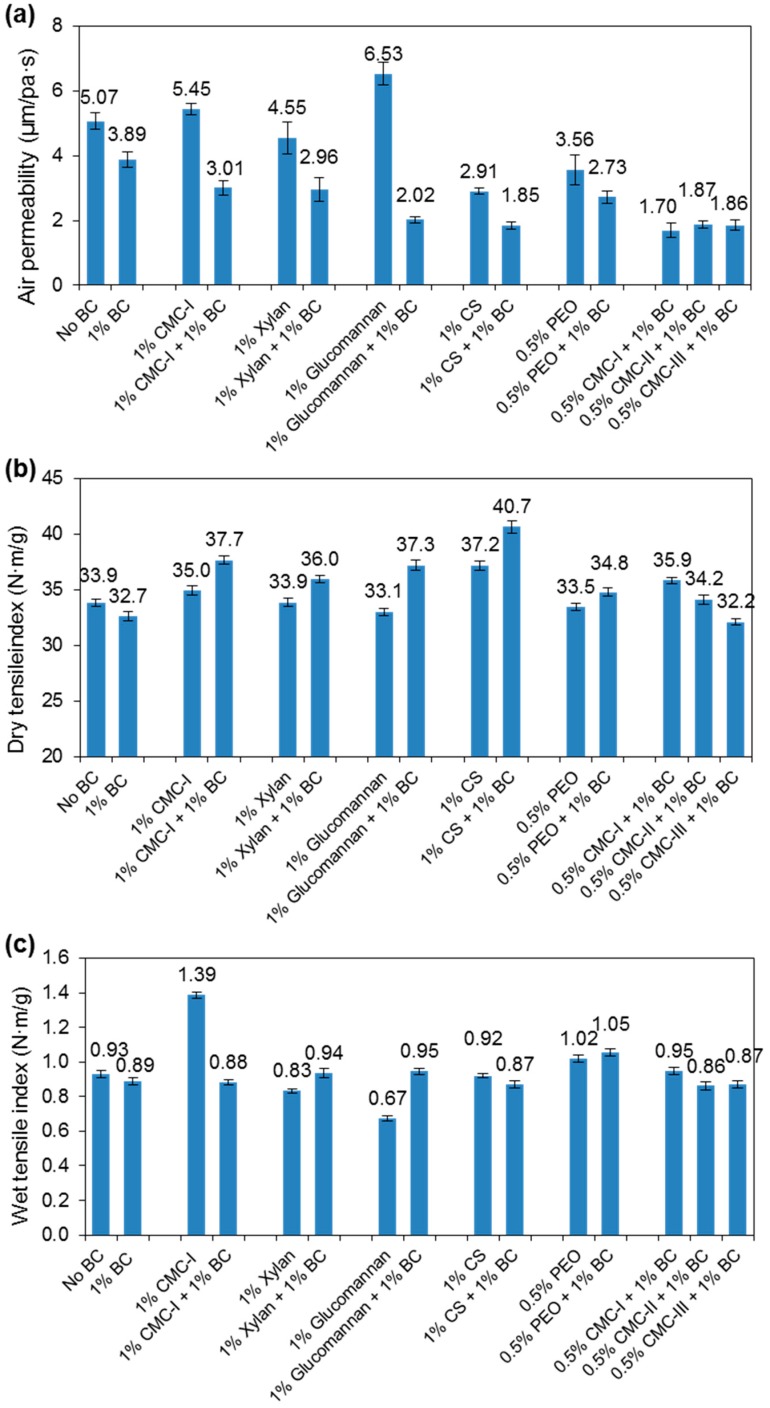
(**a**) Air permeability, (**b**) dry tensile index, and (**c**) wet tensile index of paper hand sheets produced from recycled fibers and different additive macromolecules.

Our previous studies have already proved that one of the key factors limiting the reinforcement of paper by BC fibers is the aggregation of BC fibers [12]. It was hypothesized that if BC fibers were well dispersed during sheet formation, the strength of paper sheet would be effectively improved. Therefore, to evaluate if BC fiber dispersion has positive effects on BC reinforced paper, the BC fibers dispersed by various macromolecules were added to pulps of recycled fibers to make paper sheets. The macromolecules used are CMC-I (Mw = 90,000, DS = 0.7), CMC-II (Mw = 250,000, DS = 1.2), CMC-III (Mw = 250,000, DS = 0.7), nonionic polyethylene oxide (PEO, average Mw = 4,000,000), xylan (from sugarcane bagasse, Mw = 30,000), glucomannan (from konjac), and cationized starch (DS = 0.025). The air permeability, dry tensile strength, and wet tensile strength and air permeability of BC-reinforced paper were investigated (Figure 1). Air permeability of the paper reinforced by BC may reflect the dispersion of BC to some extent. Lower air permeability indicated better BC dispersion, as found by previous study of reinforcing paper with nano-cellulose [3]. From Figure 1a, after adding dispersed BC, the air permeability of paper decreased, indicating a good dispersion of BC by macromolecules. Glucomannan and cationized starch dispersed BC gave the lowest air permeability to paper, which may correspond to their great dispersing effects to BC. From Figure 1b, Being dispersed by most of the macromolecules, the BC reinforced paper had improved dry tensile index, from 32.7 N·m/g to 37.7 N·m/g when adding CMC-I, to ~37 N·m/g when adding xylan or glucomannan, and to 40.7 N·m/g when adding cationized starch. The percentage improvement was from 15% to 25%. Without adding BC fibers, some macromolecules themselves can already improve the paper strength, such as CMC-I and cationized starch, but adding BC can further improve the tensile strength of paper. By adding only cationized starch, the paper strength had already improved to 37.2 N·m/g. Cationized starch helps to improve the retention of BC fibers or fines from recycled fibers and thus improves the paper strength [29]. The best improvement in paper strength caused by macromolecule dispersed BC was obtained with glucomannan by which an improvement of 4.2 N·m/g or 12.7% was obtained. This may correlate to that glucomannan have the best dispersing ability as demonstrated by the air permeability data (Figure 1a). From Figure 1c, the wet tensile index of paper sheet had not much improvement after adding dispersed BC, because BC fibers and recycled fibers connect through hydrogen bonding that is weak in wet. Adding only CMC improved the wet tensile index of paper a lot, which may be due to the forming of covalent bonds between CMC and plant fibers [30]. After adding BC, CMC tended to bind with BC resulting in the decrease of paper wet tensile strength. 

In sum, it can be concluded here that improved dispersion of BC fiber is helpful to improve its reinforcement to paper dry tensile strength. However, the improvement was not very high with up to ~12% caused by macromolecule-dispersed BC (taking out the reinforcement effects of macromolecules only). This showed that the quality of pulp fibers is still very important to the reinforcement effects of BC fibers. Proper dispersion of BC fibers can compensate for the pulp quality to some extent.

### 3.2. Effects of Additives on BC Fiber Dispersion

**Figure 2:**
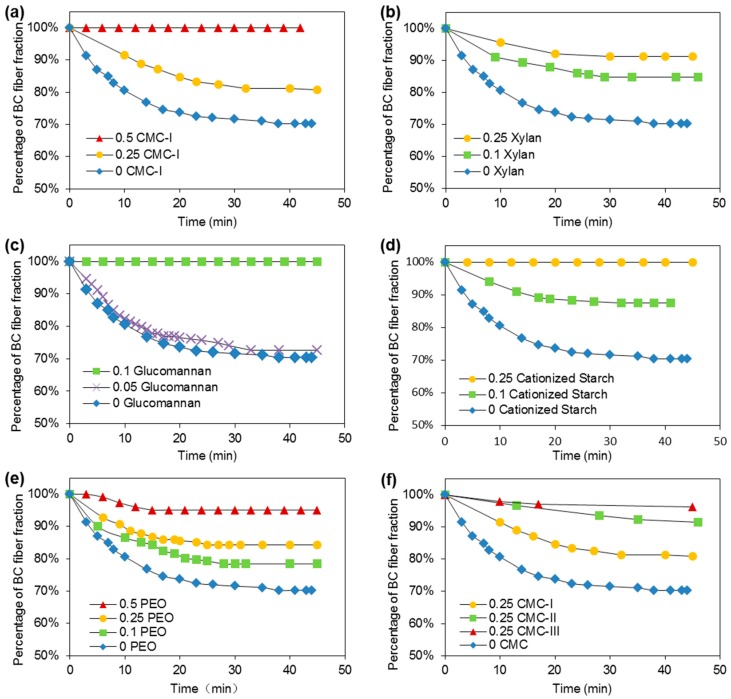
The changes of the percentage height of BC fiber fractions in the 250 mL-cylinder with time for BC fiber suspensions dispersed by (**a**) CMC-I, (**b**) xylan, (**c**) glucomannan, (**d**) cationized starch, (**e**) PEO, and (**f**) CMC.

As discussed in Section 3.1, once dispersed by various macromolecules, the BC fibers improved tensile strength of paper sheets significantly (Figure 1). However, the dispersing effects of various macromolecules on BC fiber suspension should be evaluated and correlated with paper sheet tensile strength in order to understand the mechanism for reinforcement of paper by dispersed BC. Therefore, carboxymethyl cellulose (CMC), xylan, glucomannan, cationized starch, and polyethylene oxide (PEO) were added during the disintegration of BC fibers. The total volume of BC fiber suspension was made to 250 mL (0.3 g/L) and kept in a 250 mL-cylinder. The changes of the height of BC fiber fractions with time were measured as indicator for the dispersing effects of different macromolecules (Figure 2). Adhesion of macromolecules to cellulose fiber surface may also improve the fiber dispersion through steric hindrance effects [19]. If the suspension is less stable, the BC fibers aggregate faster and thus the height of the BC fiber fraction decreases faster. From Figure 2a–e, the macromolecule leading the best colloidal stability of BC fiber suspension was glucomannan, with which the suspension had no aggregation in 50 min at 0.1 weight ratio of addition. The BC fiber suspensions with cationized starch and xylan had no aggregation of BC fiber at 0.25 weight ratio of addition, while suspensions with CMC-I and PEO had no aggregation at 0.5 weight ratio of addition. 

CMC-I has Mw = 90,000 and DS = 0.7. To evaluate the effects of molecular weight and viscosity of the macromolecules on the dispersion of BC fibers, CMC-II (Mw = 250,000, DS = 1.2) and CMC-III (Mw = 250,000, DS = 0.7) were also used to disperse BC fibers (Figure 2f). At 0.25 weight ratio of addition, CMC-III can prevent BC fibers from aggregation in 50 min, while CMC-I and -II dispersed BC fiber still had some aggregation. Therefore, the dispersing ability of CMC has an order of CMC-III > CMC-II > CMC-I. CMC-III had the same molecular weight but higher viscosity than CMC-II, and both CMC-III and -II had higher molecular weight and viscosity than CMC-I. This result postulated that, for the same macromolecule, molecular weight has positive effects on its dispersing ability for BC fibers, which could be due to the fact that high molecular weight may induce high steric hindrance. In the case of CMC with a same molecular weight, CMC-III has a lower DS than CMC-II and, thus, has more available hydroxyls. High numbers of hydroxyls induce a strong interaction with water resulting in high viscosity of CMC-III [31]. A strong interaction between CMC-III and water may result in its better dispersing effects to BC, compared to CMC-II. 

**Figure 3:**
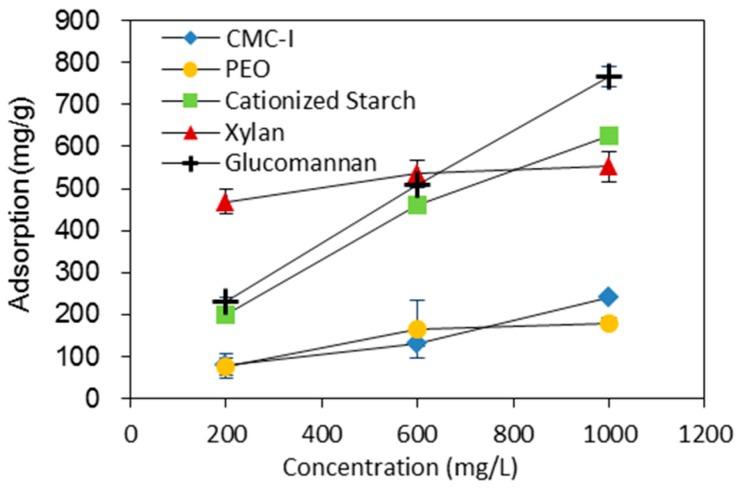
The adsorption of different macromolecules onto BC fibers at different concentrations.

**Figure 4:**
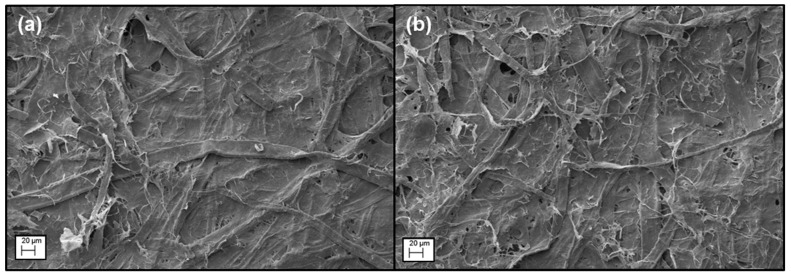
SEM images of recycled fiber paper reinforced by (**a**) undispersed BC and (**b**) glucomannan dispersed BC.

To further investigate the stabilization mechanism of BC fiber suspensions by different macromolecules, viscosity of the macromolecules (Table 1), zeta potential of BC fiber suspension (Table 2), and adsorption of macromolecules on BC fibers (Figure 3) were evaluated. The viscosities of the macromolecules were measured at 0.15 g/L, which was the same as their concentrations in BC fiber suspensions of 0.5 weight ratios. The concentration of the macromolecules in BC fiber suspension was low, resulting in very low viscosities for all the macromolecule solutions (Table 1). This indicated that viscosity was not a major factor affecting BC fiber dispersion in this case. For the zeta potential, the plain BC fiber suspension without any macromolecules added had a value of −21.2 (Table 2). By increasing the weight ratio of macromolecules to BC fibers from 0.1–0.5, the zeta potential of the BC fiber suspension with CMC-I changed from −34.2 to −60.6, with xylan changing from −21.9 to −24.3, with cationized starch from −20.0 to 29.9, and with PEO from −26.6 to −10.3 (Table 2). A high absolute value of the zeta potential usually indicates a high colloidal stability of a suspension system. However, the 0.25 weight ratio of cationized starch dispersed BC fibers had lower absolute value of zeta potential than that of 0.1 weight ratio, but had better dispersion, as shown in Figure 2; BC fiber suspension with 0.25 CMC-I had the highest zeta potential, but did not have the best colloidal stability. These phenomena indicated that there were other factors affecting the colloidal stability. Consequently, the adsorption capacity of macromolecules onto BC fibers (Figure 3) was evaluated. The adsorptions of macromolecules onto fibers were believed to have an important influence of the dispersing of cellulose fibers, since the macromolecules adsorbed by fibers can increase the steric hindrance [19]. From Figure 3, it can be seen that, at high concentration, glucomannan had the highest adsorption to BC fibers, while the adsorptions of cationized and xylan were also high. The adsorptions of CMC and PEO to BC fibers were the lowest and much smaller than other macromolecules. As shown in Figure 2, glucomannan had the best dispersing ability for BC fibers, while cationized starch and xylan were the second best. SEM images showed that glucomannan dispersed BC fibers were more evenly distributed in the paper fiber matrix compared to undispersed BC fibers (Figure 4). From Figure 3, it can be postulated that, the adsorption ability of the macromolecules onto BC fibers was very important to their dispersing effects. In another words, steric hindrance is a major factor for BC fiber dispersion. 

### 3.3. BC Fiber Dispersion by Surface Oxidation

Electrostatic repulsion is another important mechanism to improve BC fiber dispersion. TEMPO-mediated oxidation method was used to oxidize the surface of BC fibers. The C6 on anhydroglucose unit is selectively oxidized into carboxylic acid [28] and the negative charges on BC fiber surfaces may repulse each other preventing the fiber from aggregation. The oxidation was controlled at a low level avoiding the excessive decrease of the hydrogen bonds between BC fibers and plant fibers, as well as preventing the degradation of BC. BC fiber samples with different oxidized levels were numbered as OBC-2, OBC-1, and OBC-0.5, and their carboxyl contents were measured as 1.78, 1.38, and 0.41 mmol/g, respectively. 

**Figure 5:**
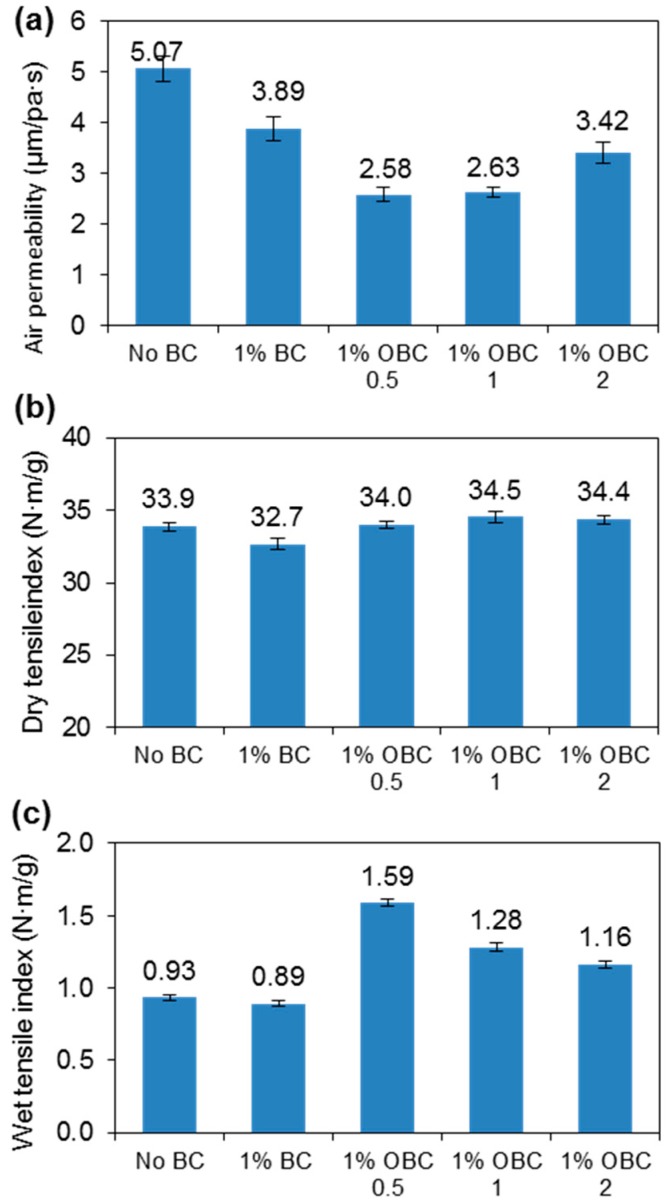
(**a**) Air permeability, (**b**) dry tensile index, and (**c**) wet tensile index of paper hand sheets produced from recycled fibers and oxidized BC fibers.

The suspensions (250 mL and 0.3 g/L) produced from all of the three oxidized BC samples did not aggregate within 50 min, indicating the TEMPO-mediated oxidation effectively improves the BC fiber dispersion or colloidal stability. The oxidized BC fibers were mixed with recycled fibers to produce reinforced paper. Figure 5a showed that the air permeability of the recycled fiber decreased by adding OBC, indicating OBC was well dispersed during paper forming [3]. At high oxidation level, the OBC reinforced paper had slightly increased air permeability may be due to the excessive degradation of BC. Figure 5b showed that adding TEMPO-mediated oxidized BC fiber does not help to improve the dry tensile index of paper. However, the wet tensile index improved greatly, especially for the BC fiber with the lowest oxidation level (OBC-0.5), i.e., from 0.89 N·m/g to 1.59 N·m/g, increased by 80% (Figure 5c). The increase in wet tensile strength indicated covalent bonds formed between fibers [32]. The explanation was that some hydroxyls were not oxidized into carboxyls, but into aldehydes [28,33], especially at low oxidation level. The aldehydes may form hemiacetals or acetals with fiber hydroxyls, leading to the improvement in paper wet strength [32]. However, the numbers of aldehyde were low and resulted in no increase in dry tensile strength, since the values of dry tensile index were high.

The oxidized BC fibers leading to limited paper strength improvement may be due to the competition of hydrogen bonds and carboxyl groups from the oxidation process. BC fibers reinforce paper by increasing hydroxyl hydrogen bonding sites between plant fibers. The carboxyl groups are introduced by the TEMPO-mediated oxidation process, and the more of the carboxyls, the better is the BC dispersion. However, carboxyls are transformed from hydroxyls. Therefore, the transformation of carboxyl groups causes the decrease of the number of hydrogen bonds between BC fibers and plant fibers, limiting the improvement of paper strength. Another major reason for limited paper strength improvement may be due to the TEMPO-mediated oxidation process degraded BC to some extent. 

In conclusion of this section, TEMPO-mediated oxidation is effective in improving the colloidal stability of BC fiber suspensions, but not effective in improving the BC fiber ability to enhance paper dry tensile index although the paper wet tensile index was improved. 

## 4. Conclusions

In this study, natural or modified polysaccharides, such as xylan, CMC, glucomannan, and cationized starch were used as additives to improve the dispersion of BC fibers or its reinforcing effects to recycled fiber paper. Good dispersion of BC fiber is helpful to its reinforcement to the dry tensile strength of paper made from recycled fiber, even though the improvement was not very high, with up to ~12% caused by macromolecule-dispersed BC. This showed that the quality of pulp fibers is still very important to the reinforcement effects of BC fibers. Proper dispersion of BC fibers can compensate for the pulp quality to some extent. 

The adsorption of the macromolecules onto BC fibers is one of the key factors for their ability to disperse BC fibers. Some macromolecules, such as cationized starch, can evidently improve the paper strength alone without adding BC due to their fiber retention ability, but its adsorption to BC fibers can still improve BC dispersion and, thus, further improve the paper strength. The dispersing effects of the macromolecules can be correlated with the tensile strength of paper sheets. Among the macromolecules studied, glucomannan had the best dispersing ability to BC fibers and led to the best paper tensile strength improvement. 

TEMPO-mediated oxidation is effective in improving the colloidal stability of BC fiber suspensions, but not effective in improving the BC fiber ability to enhance paper dry tensile index, probably because BC was degraded by the oxidation process. However, the incomplete oxidation of hydroxyls to aldehyde on BC fibers helps to improve the paper wet tensile index.

## Figures and Tables

**Table 1:** Viscosity (mean ± STDEV) of different macromolecules at 0.15 g/L concentration and measured at 250 rpm rotational speed of the rotational viscometer.

Additives	CMC-I	Xylan	Glucomannan	Cationized Starch	PEO
Viscosity (mPa·s)	30.9 ± 1.9	28.9 ± 0.6	29.4 ± 1.3	29.9 ± 0.8	33.1 ± 2.3

**Table 2:** Zeta potential (mean ± STDEV) of BC fiber suspensions with additions of different macromolecules.

Weight Ratio	Zeta potential (mV) of Macromolecule Additives					
	None	CMC-I	Xylan	Glucomannan	Cationized Starch	PEO
0.1	−21.2 ± 0.6	−34.2 ± 1.8	−22.9 ± 0.9	−23.5 ± 1.3	−20.0 ± 0.8	−26.6 ± 2.0
0.25	-	−56.8 ± 2.1	−24.9 ± 1.1	-	−13.2 ± 0.4	−13.2 ± 1.4
0.5	-	−60.6 ± 1.7	−24.3 ± 1.0	-	29.9 ± 2.2	−10.3 ± 0.7

## Abbreviations

BC	Bacterial cellulose
CMC	Carboxymethyl cellulose
OBC	Oxidized bacterial cellulose
PEO	Polyethylene oxide
